# Genital basal cell carcinoma in sun-protected sites: sex-stratified clinicopathologic features, p16/HPV status, and long-term outcomes in an Asian cohort

**DOI:** 10.3389/fmed.2026.1891922

**Published:** 2026-08-12

**Authors:** Qiuyan Duan, Yangfeng Lou, Longfei Yang, Peng Zhou

**Affiliations:** 1 Department of Dermatology, The Third Hospital of Hangzhou, Hangzhou, China; 2 Department of Urology, The Third Hospital of Hangzhou, Hangzhou, China

**Keywords:** Asian cohort, basal cell carcinoma, genital skin, human papillomavirus, p16, recurrence-free survival, sex differences, sun-protected sites

## Abstract

Basal cell carcinoma (BCC) arising in sun-protected genital sites is uncommon and lies outside the typical ultraviolet-driven model of disease. We retrospectively reviewed 41 patients with primary genital BCC treated at a tertiary referral center in China between January 2010 and December 2025, assessing clinicopathologic features, sex-stratified outcomes, p16 expression, and high-risk human papillomavirus (HPV) DNA status. The cohort included 29 males and 12 females. Female patients were older at diagnosis than male patients (74.8 ± 10.8 vs. 65.4 ± 12.0 years; *p* = 0.019). Nodular BCC was the predominant histologic subtype (29/41, 70.7%), and aggressive histologic components were present in 7 cases (17.1%). p16 immunoreactivity was detected in 4 of 37 successfully tested cases (10.8%), and high-risk HPV DNA was detected in 2 of 27 successfully tested cases (7.4%). Clinical presentation descriptors were retrospectively extracted from routine medical records and were incompletely documented. During a median follow-up of 51 months, five patients developed local recurrence and one male patient developed inguinal lymph node metastasis. In this exploratory cohort, no statistically significant sex-associated difference in recurrence-free survival was detected (log-rank *p* = 0.65), but the small number of events precluded inference of equivalent outcomes or exclusion of clinically meaningful sex-related differences. In this Asian cohort, genital BCC was usually nodular, only infrequently associated with detectable high-risk HPV in available tested specimens, and generally favorable after surgery. Because genital lesions may be concealed and clinically non-specific, biopsy should be considered for persistent or suspicious genital lesions, with ongoing postoperative surveillance.

## Introduction

Basal cell carcinoma (BCC) is usually understood through the lens of ultraviolet exposure. Genital BCC, however, arises in largely sun-protected skin and therefore raises a distinct clinical question: whether its presentation, viral-marker profile, and long-term course differ from those of conventional sun-exposed BCC. Because these tumors occur in concealed anatomic sites, they may be overlooked during routine examination or managed empirically as inflammatory, infectious, or squamous lesions before biopsy, leaving their clinicopathologic spectrum and prognosis incompletely defined (1, 2).

The evidence base remains limited. Most available publications consist of isolated case reports, small institutional series, systematic reviews with heterogeneous case-level data, or population-based analyses that cannot combine detailed histologic subtyping, p16/high-risk human papillomavirus (HPV) assessment, sex-stratified comparison, and recurrence-level follow-up in the same cohort (3, 4). Published Asian data appear particularly limited. Before the present study, the Asian literature consisted mainly of isolated case reports and our previously reported 12-patient male-only institutional series of scrotal BCC (5). To our knowledge, no Asian cohort has previously combined both sexes, centralized histopathologic review, p16/high-risk HPV testing, and recurrence-level follow-up in a single study. Whether sex-related patterns described in conventional BCC extend to genital sites, and whether high-risk HPV has any meaningful role in tumor biology, remain unresolved (6–8).

We previously reported a preliminary institutional series of 12 male patients with scrotal BCC treated between January 2012 and December 2022 (5). Under the current inclusion and exclusion criteria, all 12 previously published cases were re-screened and qualified for inclusion in the present expanded cohort. The current study therefore extends, rather than duplicates, that prior experience by adding female genital BCC cases, broadening eligible sun-protected genital sites, extending outcome ascertainment through December 2025, and incorporating a sex-stratified and viral-marker focus. The present manuscript should be read as an expanded institutional cohort rather than as an independent male-only series. We used this cohort to define the clinicopathologic spectrum of genital BCC, explore sex-stratified presentation and outcomes, and estimate the frequency of detectable p16 and high-risk HPV DNA in available tissue.

## Materials and methods

### Study design and patients

**Figure 1:**
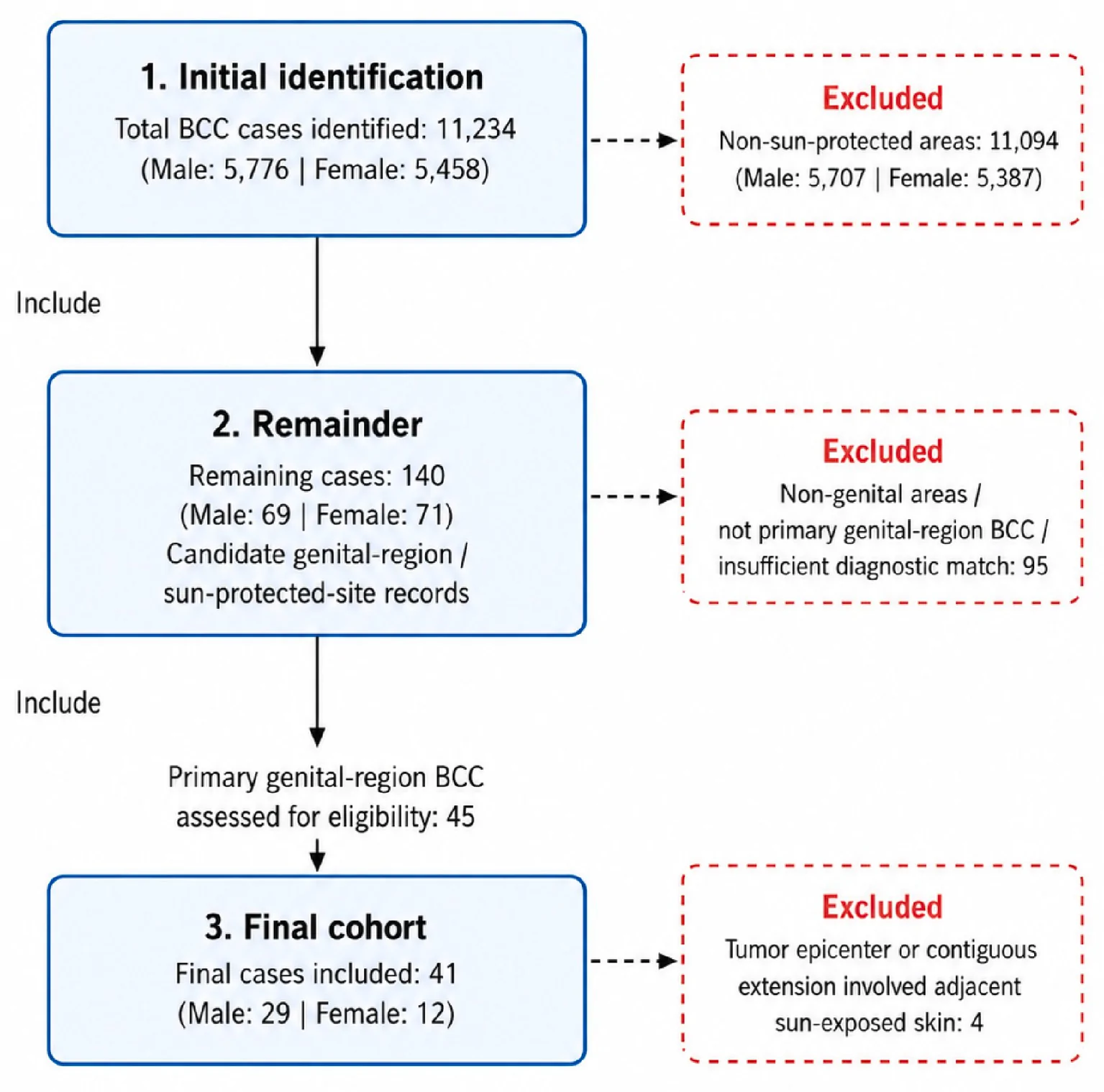
Flow diagram of case identification and patient selection. Institutional pathology records with a diagnosis of basal cell carcinoma between January 2010 and December 2025 were screened. Records arising outside candidate genital-region or sun-protected sites, records not representing histopathologically confirmed primary genital-region BCC, records lacking sufficient baseline or follow-up information, and tumors extending into adjacent sun-exposed skin were excluded. The final analytic cohort included 41 patients with primary BCC arising in sun-protected genital sites. All 12 male scrotal BCC cases from the previously published institutional series were re-screened under the current eligibility criteria and included in this expanded cohort.

This retrospective study was approved by the Ethics Committee of Hangzhou Third People’s Hospital (approval No. 2026-KA075) and followed the Declaration of Helsinki and STROBE reporting recommendations. We searched institutional clinical and pathology databases for patients treated between January 2010 and December 2025 for primary BCC arising in sun-protected genital sites. Eligible sites included the scrotum, penis, vulva, labia, perineum, and adjacent non-sun-exposed genital skin. Pathology database searches combined “basal cell carcinoma” with genital anatomic site terms and were cross-checked against clinical records. We first identified 11,234 pathology records with a diagnosis of BCC during the study period. After exclusion of 11,094 records arising outside candidate genital-region or sun-protected sites, 140 candidate records were reviewed. Ninety-five records were excluded because they did not represent histopathologically confirmed primary genital-region BCC or lacked sufficient baseline/follow-up information. Forty-five primary genital-region BCC cases were assessed for eligibility, and 4 were excluded because the tumor epicenter or contiguous extension involved adjacent sun-exposed skin. The final analytic cohort included 41 patients with primary BCC arising in sun-protected genital sites (Figure 1).

Eligible cases required histopathologically confirmed primary genital BCC, tumor origin in a sun-protected genital site, and sufficient clinical, histopathologic, and follow-up data. We excluded tumors extending into adjacent sun-exposed skin, recurrent tumors referred after treatment elsewhere without complete baseline information, and cases lacking sufficient diagnostic or follow-up information. The exclusion of tumors extending into adjacent sun-exposed skin was prespecified to preserve a biologically and anatomically focused cohort of sun-protected genital BCC, although its potential selection effect was considered in the limitations.

Cases from our previously published scrotal BCC series were not automatically carried forward. Each candidate case was re-screened under the present eligibility criteria using pathology accession identifiers, diagnosis dates, anatomic site, and clinical records. All 12 cases from the prior male-only scrotal BCC series met the present inclusion criteria and were included in this expanded cohort.

**Table 1:** Clinicopathologic characteristics and outcomes stratified by sex.

Variable	Overall (*N* = 41)	Male (*n* = 29)	Female (*n* = 12)	*p-*value
Age, mean ± SD (years)	68.2 ± 12.3	65.4 ± 12.0	74.8 ± 10.8	0.019
Tumor size, mean ± SD (cm)	1.8 ± 0.9	1.7 ± 0.8	2.1 ± 1.1	0.21
Predominant broad anatomical region, *n* (%)	—	Scrotum 25 (86.2)	Labia majora 11 (91.7)	—
Nodular subtype, *n* (%)	29 (70.7)	20 (69.0)	9 (75.0)	0.72
Aggressive histologic features, *n* (%)	7 (17.1)	5 (17.2)	2 (16.7)	1.000
p16 IHC positive, n/*N* (%)	4/37 (10.8)	3/26 (11.5)	1/11 (9.1)	0.82
High-risk HPV DNA positive, n/*N* (%)	2/27 (7.4)	1/19 (5.3)	1/8 (12.5)	0.48
Treatment: wide local excision / Mohs / other surgical management, *n* (%)	35 (85.4) / 4 (9.8) / 2 (4.9)	26 (89.7) / 2 (6.9) / 1 (3.4)	9 (75.0) / 2 (16.7) / 1 (8.3)	0.35
Local recurrence, *n* (%)	5 (12.2)	3 (10.3)	2 (16.7)	0.620
Inguinal lymph node metastasis, *n* (%)	1 (2.4)	1 (3.4)	0	1.000
Median follow-up, months	51	52	48	0.68
Follow-up range, months	—	6–126	8–109	—
Estimated 3-year RFS, %	92.7	93.1	91.7	0.650
Estimated 5-year RFS, %	87.8	89.7	83.3	—

**Figure 2:**
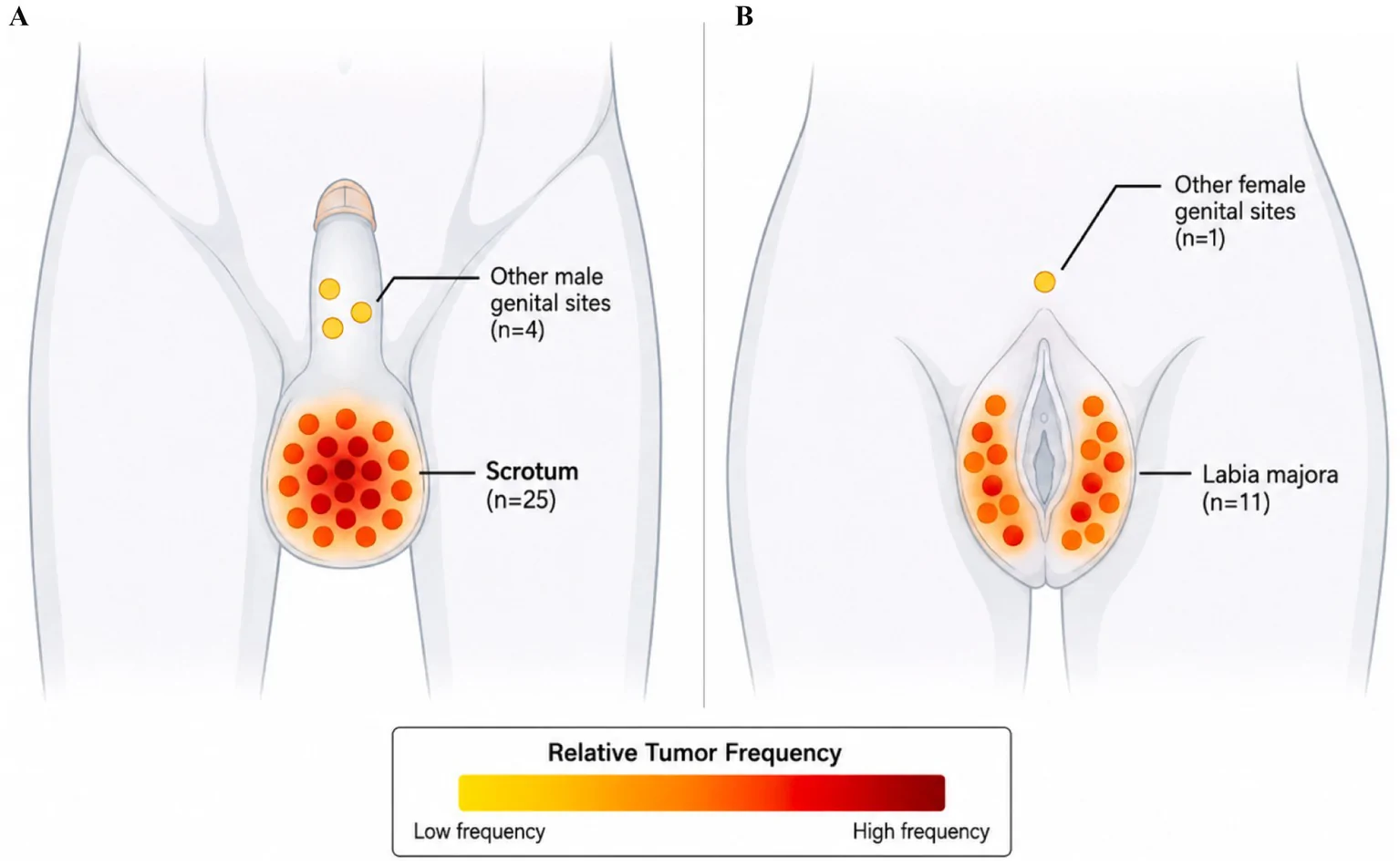
Anatomical distribution of genital basal cell carcinoma according to sex. The anatomical schematic was prepared as an illustrative diagram and does not contain patient-identifiable information. **(A)** Distribution of male genital BCC cases. Scrotal involvement represented the predominant broad anatomical region in male patients, accounting for 25 of 29 cases; the remaining 4 cases involved other male genital sites. **(B)** Distribution of female genital BCC cases. Labia majora involvement represented the predominant broad anatomical region in female patients, accounting for 11 of 12 cases; the remaining 1 case involved another female genital site. Anatomical sites are presented as broad regions to maintain consistency with Table 1.

HPV, human papillomavirus; IHC, immunohistochemistry; RFS, recurrence-free survival; SD, standard deviation. Aggressive histologic features were defined as infiltrative, micronodular, or morpheaform components. The *p*-value for recurrence-free survival is from the log-rank test. p16 and high-risk HPV percentages were calculated among successfully tested cases only. Other surgical management indicates surgery not classified as wide local excision or Mohs micrographic surgery in the extracted dataset. Anatomical sites were summarized by the predominant broad anatomical region to ensure consistency between Table and Figure 2. Less common sites were grouped as other genital sites because detailed subsite classification was not used for formal statistical comparison.

Medical records were reviewed for age at diagnosis, sex, lesion location, clinical morphology, tumor size, predisposing factors, histopathologic subtype, treatment modality, and outcome. Clinical presentation variables were abstracted retrospectively from routine medical records, including lesion duration, symptoms, morphology, pigmentation, ulceration, discharge, prior treatment, and anatomic subsite when documented. Because these features were not collected using a standardized prospective instrument, denominators varied by variable and clinical descriptors were analyzed descriptively rather than as formal comparative endpoints.

### Histopathologic evaluation

Formalin-fixed biopsy and resection specimens were routinely processed, paraffin embedded, sectioned, and stained with hematoxylin and eosin. Two experienced dermatopathologists independently reviewed all available slides. Tumors were classified according to World Health Organization criteria as nodular, superficial, infiltrative, micronodular, morpheaform, or mixed. Aggressive histologic features were defined by infiltrative, micronodular, or morpheaform components.

### p16 immunohistochemistry and HPV DNA testing

p16 immunohistochemistry and high-risk HPV DNA testing were performed when adequate archived tissue was available. Formalin-fixed paraffin-embedded sections were cut at 4 μm and stained using the Maixin immunohistochemical platform (Fuzhou Maixin Biotech Co., Ltd., China) according to standard institutional protocols. After deparaffinization and rehydration, heat-induced epitope retrieval was performed in EDTA buffer using a microwave-based retrieval procedure. Endogenous peroxidase activity was blocked, and sections were incubated with a ready-to-use mouse monoclonal anti-p16 antibody (clone MX007; product no. MAB-0673; Fuzhou Maixin Biotech Co., Ltd., China) according to the manufacturer’s instructions and the locally validated laboratory protocol. Detection was performed using a polymer-based visualization system with diaminobenzidine as the chromogen, followed by hematoxylin counterstaining.

Two experienced dermatopathologists independently evaluated p16 staining. p16 was considered positive only when tumor cells showed strong, diffuse block-type nuclear and cytoplasmic staining involving ≥70% of tumor cells. Focal, weak, or patchy staining was classified as negative for the primary analysis. Appropriate positive and negative controls were included in each staining run.

High-risk HPV DNA testing was performed on formalin-fixed paraffin-embedded tissue using a biochip-based nucleic acid detection kit with a nucleic acid chip detector (model BHF-VI; Bohui, China), according to the manufacturer’s instructions and the institutional molecular pathology workflow. Tissue DNA was extracted either immediately after sampling or after storage at −70 °C until analysis. The assay covered HPV16, 18, 31, 33, 35, 39, 45, 51, 52, 53, 56, 58, 59, 66, 68, 73, and 82. A valid result required successful internal control amplification. Cases with inadequate residual tissue, insufficient DNA quality, or failed internal control amplification were excluded from HPV status analysis. Positivity rates for p16 and high-risk HPV DNA were calculated among successfully tested cases only.

### Treatment and follow-up

All patients underwent surgery as primary treatment. Local recurrence was defined as histologically confirmed tumor reappearance at or adjacent to the original surgical site. Recurrence-free survival was measured from primary surgery to first local recurrence or last follow-up.

### Statistical analysis

Continuous variables are reported as mean ± standard deviation or median with range, as appropriate, and were compared using the independent-samples *t* test or Mann–Whitney *U* test according to distribution. Categorical variables were analyzed using Fisher’s exact test. p16 and high-risk HPV positivity rates were calculated among successfully tested cases only.

Recurrence-free survival (RFS) was measured from primary surgery to first histologically confirmed local recurrence or last follow-up. RFS was estimated by the Kaplan–Meier method and compared by the log-rank test. Because the female subgroup and the number of recurrence events were small, all sex-stratified outcome analyses were treated as exploratory and descriptive. The absence of a statistically significant log-rank result was not interpreted as evidence of equivalence or absence of clinically meaningful sex-related differences. Multivariable time-to-event modeling was not performed because the sparse number of recurrence events would not support stable adjusted estimates. A two-sided *p-*value < 0.05 was considered statistically significant.

## Results

The cohort included 41 patients with primary BCC arising in sun-protected genital sites: 29 males and 12 females. The predominant broad anatomical regions were the scrotum in male patients and the labia majora in female patients (Figure 2). Details of case-level reconciliation between the previously published 12-patient male-only scrotal BCC series and the present expanded cohort are provided in Supplementary Table S2. All 12 previously reported cases were re-screened under the current eligibility criteria and included in the present cohort. Four otherwise eligible genital-region BCC cases were excluded because the tumor epicenter or contiguous extension involved adjacent sun-exposed skin; the characteristics of these excluded cases are summarized in Supplementary Table S3.

Female patients were older at diagnosis than male patients (74.8 ± 10.8 vs. 65.4 ± 12.0 years; *p* = 0.019). Nodular BCC was the predominant histologic subtype (29/41, 70.7%) and did not differ significantly by sex. Other tumors showed superficial, infiltrative, micronodular, morpheaform, or mixed patterns. Aggressive histologic components were present in 7 cases (17.1%).

Because residual tissue availability varied, viral-marker denominators differed between assays. p16 immunohistochemistry was positive in 4 of 37 successfully tested cases (10.8%), and high-risk HPV DNA was positive in 2 of 27 successfully tested cases (7.4%). Neither marker showed a clear sex-related distribution.

**Figure 3:**
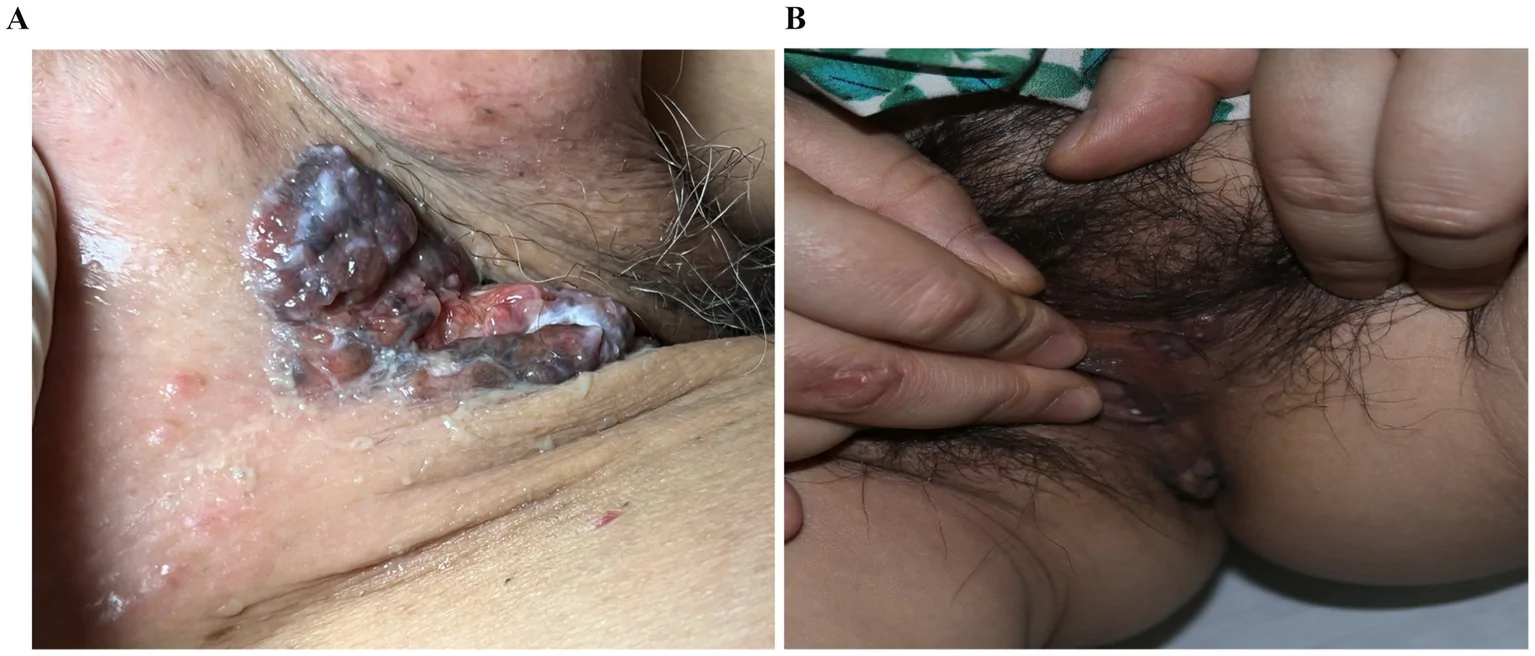
Representative clinical images of primary basal cell carcinoma in sun-protected genital sites. **(A)** Grey-pink ulcerated nodule with greyish-white discharge on the right scrotum. **(B)** Multifocal hyperpigmented and verrucous nodules involving the labia, with focal ulceration and greyish-white discharge.

Clinical morphology was available from routine medical records but was not captured using a standardized prospective instrument. When documented, lesions were described as nodular, ulcerated, pigmented, verrucous, plaque-like, or discharge-associated. Because documentation was incomplete and non-standardized, these descriptors were used to provide clinical context rather than formal statistical comparison. Descriptive clinical presentation features are summarized in Supplementary Table S4, and representative clinical presentations are shown in Figure 3. Among cases with documented records, nodular morphology was noted in 25 of 37 cases, ulceration in 18 of 38 cases, and prior empirical treatment in 29 of 41 cases.

**Figure 4:**
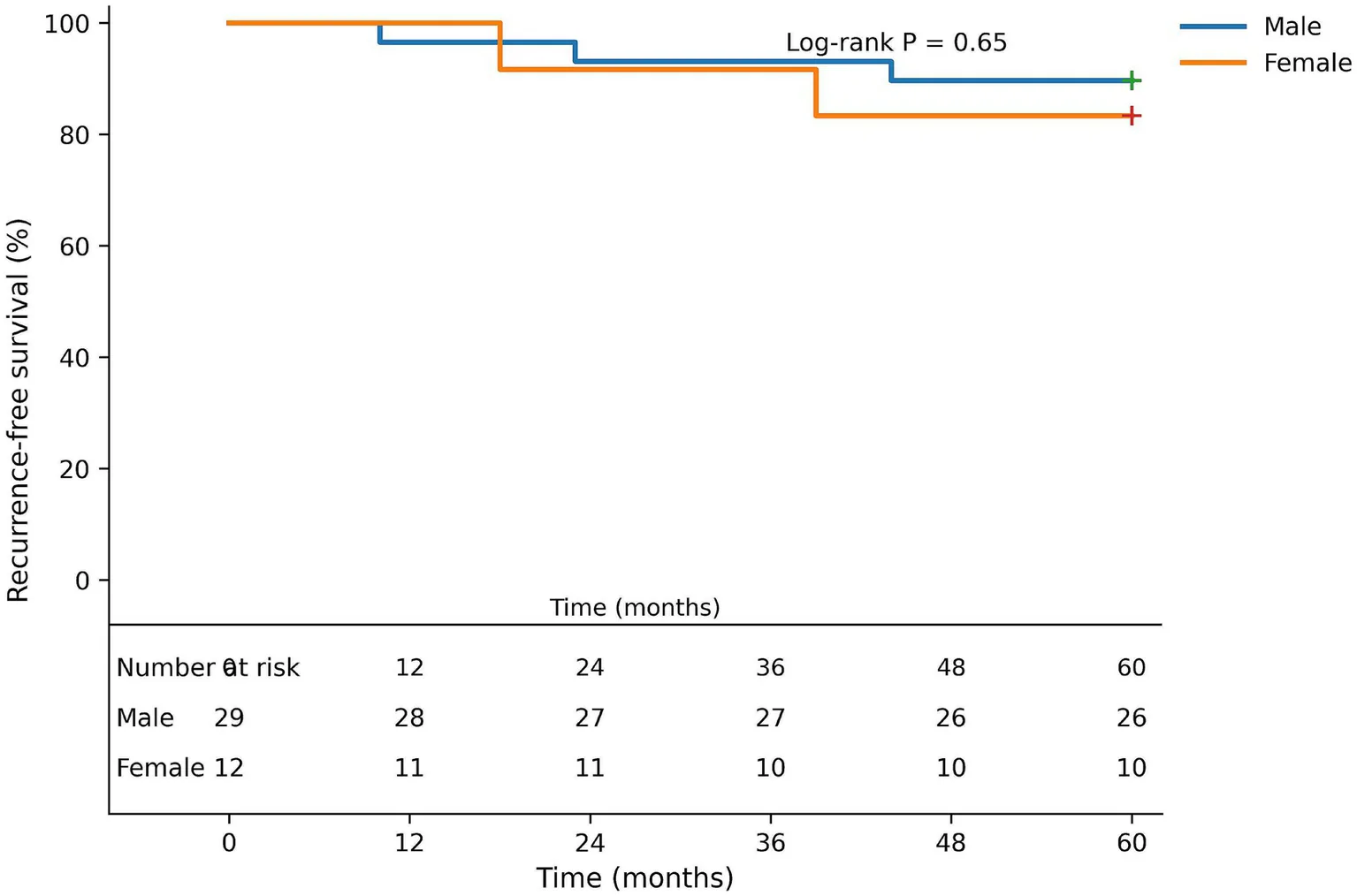
Exploratory Kaplan–Meier analysis of recurrence-free survival according to sex. Recurrence-free survival (RFS) was estimated using the Kaplan–Meier method and compared between male and female patients with the log-rank test. Local recurrence occurred in 3 male patients at 10, 23, and 44 months and in 2 female patients at 18 and 39 months. No statistically significant sex-associated difference in RFS was detected in this exploratory analysis (log-rank *p* = 0.65). Because only 5 local recurrence events occurred, these data should not be interpreted as evidence of equivalent outcomes between male and female patients. The number-at-risk table is shown below the plot.

Median follow-up was 51 months. Five patients developed local recurrence: three males at 10, 23, and 44 months and two females at 18 and 39 months. One male patient developed inguinal lymph node metastasis. Event-level outcomes are summarized in Supplementary Table S1. Exploratory Kaplan–Meier analysis did not detect a statistically significant sex-associated difference in recurrence-free survival (log-rank *p* = 0.65). However, the analysis was underpowered because only five local recurrences occurred, and these data should not be interpreted as evidence of equivalent male and female outcomes or as excluding clinically meaningful sex-related differences. Estimated 3-year recurrence-free survival was 93.1% in males and 91.7% in females; the corresponding 5-year rates were 89.7% and 83.3%, respectively (Figure 4).

## Discussion

The central observation from this expanded institutional cohort is that genital BCC in sun-protected sites behaved largely as an indolent, nodular-predominant tumor in this Asian population. Female patients were older at diagnosis. In exploratory survival analysis, we did not detect a statistically significant sex-associated difference in recurrence-free survival, but the small female subgroup and limited number of recurrence events preclude any conclusion of equivalent outcomes and cannot exclude clinically meaningful sex-related differences. p16 immunoreactivity and high-risk HPV DNA were uncommon in tested specimens, and outcomes after surgery were generally favorable.

These data refine, rather than simply repeat, the existing literature. Population-based analyses of external genital BCC have clarified epidemiology but generally lack granular histologic, viral-marker, and recurrence-level data (3). Recent molecular work on BCC arising at sun-protected sites has described indolent behavior and PTCH1 alterations without dominant ultraviolet mutational signatures, a pattern broadly consistent with the low-recurrence profile observed here (6). Scrotal BCC reviews and vulvar series likewise support the rarity and usually favorable course of these tumors, although published cases remain limited and heterogeneously reported (4, 8). The present study extends our prior 12-patient male-only scrotal BCC series by incorporating female cases, broader sun-protected genital sites, longer follow-up, centralized review, and p16/high-risk HPV assessment. When compared with non-Asian and international reports, several findings from the present Asian cohort appear broadly concordant with the existing literature. Previous population-based analyses and case-based reviews of external genital or scrotal BCC have consistently emphasized the rarity of this tumor subset, its predominance in older adults, and its generally favorable clinical course after adequate surgical treatment. Similarly, in our cohort, genital BCC arising in sun-protected sites was usually nodular in histology and showed a low frequency of recurrence during long-term follow-up. These observations suggest that, despite geographic and ethnic differences, genital BCC may share several clinicopathologic features across populations.

Nevertheless, direct comparison between Asian and non-Asian cohorts remains limited by heterogeneity in study design, anatomical definitions, follow-up duration, and availability of pathological or molecular data. Many previously published reports from non-Asian populations were based on population registries, isolated case reports, or systematic reviews of heterogeneous case-level data, whereas detailed information on p16 expression, high-risk HPV DNA status, centralized histopathological review, and recurrence timing was often unavailable. The present study therefore adds complementary Asian cohort-level evidence and suggests that high-risk HPV is not frequently detected in available genital BCC specimens. However, because viral testing was incomplete and the number of outcome events was small, these comparisons should be interpreted cautiously. Larger multicenter studies across different geographic regions will be needed to determine whether true ethnic or regional differences exist in the biology, presentation, or prognosis of genital BCC.

The older age at diagnosis in female patients is better interpreted as a signal of presentation than as evidence of greater biologic aggressiveness. Concealed genital lesions may remain subtle, be difficult for patients to recognize, or be treated empirically as inflammatory dermatoses, infection, or squamous neoplasia before biopsy. Local hormonal or tissue factors could also contribute, but this cohort cannot resolve that mechanism.

The viral-marker profile has practical diagnostic relevance. Genital BCC can mimic, or be considered alongside, HPV-driven anogenital squamous carcinoma, basaloid squamous neoplasia, adnexal tumors, and other penile or vulvar malignancies (9, 10). Contemporary reviews emphasize the multifactorial risk landscape of penile cancer, including HPV infection, smoking, chronic inflammatory conditions, hygiene-related factors, and socioeconomic determinants (10). In our tested BCC cases, block-type p16 immunoreactivity and high-risk HPV DNA were infrequent, arguing against a frequent detectable high-risk HPV association in available specimens. This conclusion must remain cautious: testing was incomplete, tissue availability may have introduced selection bias, and formalin-fixed tissue can affect assay sensitivity. The data therefore indicate low detection in available specimens, not definitive exclusion of viral involvement.

The clinical message is practical rather than frequency-based. Genital BCC may clinically overlap with inflammatory dermatoses, infection, squamous neoplasia, or adnexal tumors; therefore, biopsy should be considered for nonhealing or clinically suspicious genital lesions rather than prolonged empirical therapy. Surgery remains the mainstay of treatment (11). In anatomically and functionally sensitive sites, tissue-sparing approaches such as Mohs micrographic surgery may help achieve margin control while limiting morbidity. Late recurrences beyond 3 years in this cohort support structured surveillance even when the initial course appears indolent.

This study has several strengths: a relatively large sample for a rare tumor site, centralized histopathologic review, inclusion of both sexes, viral-marker assessment, and extended follow-up. Its limitations are equally important. The retrospective single-center design, modest female subgroup, only five recurrence events, incomplete p16 and HPV testing, incomplete margin and operative variables, non-standardized documentation of clinical morphology, and lack of comprehensive molecular profiling restrict inference. Exclusion of tumors extending into adjacent sun-exposed skin was necessary to preserve the focus on sun-protected genital BCC, but this may have selected for a more anatomically restricted subset and may limit generalizability to broader external genital-region BCC. Referral patterns at a tertiary center in China may also limit generalizability. These constraints support the need for multicenter studies with standardized clinical morphology documentation, viral testing, margin data, case-level recurrence reporting, and molecular analyses.

In conclusion, BCC arising in sun-protected genital sites appears to be an uncommon but generally indolent subset of non-sun-exposed BCC in this cohort. The findings support prompt biopsy of nonhealing or clinically suspicious genital lesions in older adults, adequate excision with attention to function and cosmesis, and continued surveillance after treatment. Sex-stratified outcome findings and viral-marker results should be interpreted cautiously because of the small number of events, incomplete HPV testing, and non-standardized clinical presentation documentation.

## Data Availability

The original contributions presented in the study are included in the article/Supplementary material, further inquiries can be directed to the corresponding author.
