# Supplementary material for: Genital basal cell carcinoma in sun-protected sites: sex-stratified clinicopathologic features, p16/HPV status, and long-term outcomes in an Asian cohort
Source: Front Med (Lausanne). 2026 Aug 12;13:1891922. doi: 10.3389/fmed.2026.1891922 (PMC13506306; doi:10.3389/fmed.2026.1891922)
Supplement: Supplementary file 1 [file Table_1.docx]

**SUPPLEMENTARY MATERIAL**

**Supplementary Tables S1-S4**

*Genital basal cell carcinoma in sun-protected sites: sex-stratified clinicopathologic features, p16/HPV status, and long-term outcomes in an Asian cohort*

**Qiuyan Duan, Yangfeng Lou, Longfei Yang, and Peng Zhou**

Frontiers in Medicine | DOI: 10.3389/fmed.2026.1891922

This file contains Supplementary Tables S1-S4 cited in the article. All case identifiers are study-specific and non-identifying.

# Supplementary Table S1. Event-level summary of recurrence and metastasis

| **Event case** | **Sex** | **Event type** | **Timing after surgery** | **Comment** |
| --- | --- | --- | --- | --- |
| 1 | Male | Local recurrence | 10 months | Histologically confirmed recurrence at or adjacent to the original surgical site |
| 2 | Male | Local recurrence | 23 months | Histologically confirmed recurrence at or adjacent to the original surgical site |
| 3 | Male | Local recurrence | 44 months | Late recurrence supporting long-term surveillance |
| 4 | Female | Local recurrence | 18 months | Histologically confirmed recurrence at or adjacent to the original surgical site |
| 5 | Female | Local recurrence | 39 months | Late recurrence supporting long-term surveillance |
| 6 | Male | Inguinal lymph node metastasis | Not available in the extracted dataset | Managed with excision and inguinal lymphadenectomy; no recurrence documented after nodal treatment. |

# Supplementary Table S2. Case-level reconciliation between the previously published institutional scrotal BCC series and the present expanded genital BCC cohort

| **Prior published case ID** | **Current cohort case ID** | **Sex** | **Site in prior report** | **Prior series period** | **Re-screened under current criteria** | **Included in present cohort** |
| --- | --- | --- | --- | --- | --- | --- |
| Prior case 1 | Current case M01 | Male | Scrotum | 2012-2022 | Yes | Yes |
| Prior case 2 | Current case M02 | Male | Scrotum | 2012-2022 | Yes | Yes |
| Prior case 3 | Current case M03 | Male | Scrotum | 2012-2022 | Yes | Yes |
| Prior case 4 | Current case M04 | Male | Scrotum | 2012-2022 | Yes | Yes |
| Prior case 5 | Current case M05 | Male | Scrotum | 2012-2022 | Yes | Yes |
| Prior case 6 | Current case M06 | Male | Scrotum | 2012-2022 | Yes | Yes |
| Prior case 7 | Current case M07 | Male | Scrotum | 2012-2022 | Yes | Yes |
| Prior case 8 | Current case M08 | Male | Scrotum | 2012-2022 | Yes | Yes |
| Prior case 9 | Current case M09 | Male | Scrotum | 2012-2022 | Yes | Yes |
| Prior case 10 | Current case M10 | Male | Scrotum | 2012-2022 | Yes | Yes |
| Prior case 11 | Current case M11 | Male | Scrotum | 2012-2022 | Yes | Yes |
| Prior case 12 | Current case M12 | Male | Scrotum | 2012-2022 | Yes | Yes |

Abbreviations: BCC, basal cell carcinoma; HPV, human papillomavirus.

Footnote: All 12 cases from the previously published male-only institutional scrotal BCC series were individually re-screened under the current inclusion and exclusion criteria using pathology accession information, diagnosis date, anatomic site, and clinical records. All 12 cases met the present eligibility criteria because they were histopathologically confirmed primary scrotal BCCs arising in sun-protected genital sites with sufficient clinical, histopathologic, and follow-up data. They were therefore included in the current expanded cohort. The present study adds information beyond the prior report by broadening the study period to January 2010-December 2025, including female genital BCC cases, expanding eligible sun-protected genital sites, extending outcome ascertainment, and incorporating sex-stratified analyses and p16/high-risk HPV assessment when tissue was available.

# Supplementary Table S3. Characteristics of genital-region BCC cases excluded because of extension into adjacent sun-exposed skin

| **Excluded case ID** | **Sex** | **Age, years** | **Anatomic site** | **Adjacent sun-exposed skin** | **Main histologic subtype** | **Reason for exclusion** | **Outcome after exclusion** | **Comment** |
| --- | --- | --- | --- | --- | --- | --- | --- | --- |
| Excluded case 1 | Male | 74 | Scrotum extending to right inguinal fold | Yes (inguinal fold/lower abdomen) | Nodular | Tumor epicenter or contiguous extension involved adjacent sun-exposed skin | None | Excluded to preserve the sun-protected genital-site definition |
| Excluded case 2 | Female | 68 | Left labia majora extending to mons pubis | Yes (mons pubis/groin crease) | Superficial | Tumor epicenter or contiguous extension involved adjacent sun-exposed skin. | None | Excluded to preserve the sun-protected genital-site definition. |
| Excluded case 3 | Male | 79 | Penile base extending to suprapubic region | Yes (suprapubic skin) | Infiltrative | Tumor epicenter or contiguous extension involved adjacent sun-exposed skin. | None | Excluded to preserve the sun-protected genital-site definition. |
| Excluded case 4 | Female | 71 | Vulvar/perineal region extending to upper inner thigh | Yes (inner thigh skin) | Nodular | Tumor epicenter or contiguous extension involved adjacent sun-exposed skin. | Local recurrence at 24 months (re-excised, no metastasis) | Excluded to preserve the sun-protected genital-site definition. |

Footnote: These 4 cases were excluded from the final analytic cohort because the tumor epicenter or contiguous extension involved adjacent sun-exposed skin. They are shown separately to clarify the exclusion process and to acknowledge the potential selection effect introduced by restricting the cohort to BCC arising in sun-protected genital sites.

# Supplementary Table S4. Descriptive clinical presentation features when documented (N = 41 total cases)

| **Feature** | **Documented cases, n/N (%)** |
| --- | --- |
| Nodular lesion | 25/37 (67.6%) |
| Ulceration | 18/38 (47.4%) |
| Pigmentation | 12/35 (34.3%) |
| Verrucous appearance | 9/36 (25.0%) |
| Discharge-associated lesion | 11/34 (32.4%) |
| Plaque-like lesion | 7/33 (21.2%) |
| Prior empirical treatment | 29/41 (70.7%) |
| Documented lesion duration | 31/41 (75.6%) |

Footnote: Percentages are calculated among cases in which the specific feature was explicitly documented or assessable in the available medical records (denominators <41 reflect incomplete documentation, consistent with the retrospective, non-standardized data collection described in Methods). Morphology categories are not mutually exclusive. For documented lesion duration: median 5 months (range 1-36 months); 24/31 (77%) were persistent (>=3 months). Prior empirical treatment mainly included topical/systemic antibiotics, antifungals, or corticosteroids for presumed infectious or inflammatory conditions.
